# Characterization of recovery, repair, and inflammatory processes following contusion spinal cord injury in old female rats: is age a limitation?

**DOI:** 10.1186/1742-4933-11-15

**Published:** 2014-10-29

**Authors:** Mitra J Hooshmand, Manuel D Galvan, Elizabeth Partida, Aileen J Anderson

**Affiliations:** 1 Institute for Memory Impairments and Neurological Disorders, University of California Irvine, 2001 Sue and Bill Gross Stem Cell Research, Irvine, CA 92697-4292, USA; 2 Sue and Bill Gross Stem Cell Research Center, University of California Irvine, Irvine, CA 92697, USA; 3 Reeve-Irvine Research Center, University of California Irvine, Irvine, CA 92697, USA; 4 Anatomy and Neurobiology, University of California Irvine, Irvine, CA 92697, USA

**Keywords:** Aging, Spinal cord injury, Inflammation, Locomotor function, Complement

## Abstract

**Background:**

Although the incidence of spinal cord injury (SCI) is steadily rising in the elderly human population, few studies have investigated the effect of age in rodent models. Here, we investigated the effect of age in female rats on spontaneous recovery and repair after SCI. Young (3 months) and aged (18 months) female rats received a moderate contusion SCI at T9. Behavioral recovery was assessed, and immunohistocemical and stereological analyses performed.

**Results:**

Aged rats demonstrated greater locomotor deficits compared to young, beginning at 7 days post-injury (dpi) and lasting through at least 28 dpi. Unbiased stereological analyses revealed a selective increase in percent lesion area and early (2 dpi) apoptotic cell death caudal to the injury epicenter in aged versus young rats. One potential mechanism for these differences in lesion pathogenesis is the inflammatory response; we therefore assessed humoral and cellular innate immune responses. No differences in either acute or chronic serum complement activity, or acute neutrophil infiltration, were observed between age groups. However, the number of microglia/macrophages present at the injury epicenter was increased by 50% in aged animals versus young.

**Conclusions:**

These data suggest that age affects recovery of locomotor function, lesion pathology, and microglia/macrophage response following SCI.

## Background

Studies of human spinal cord injury (SCI) cases suggest that the role of age in recovery and mortality is complex [1-3]. A number of confounding variables impact age outcomes after SCI including, gender, severity, level of injury, and preexisting medical co-morbidities [4]. Thus, the effect of age-associated processes on recovery, repair, and mortality in the clinical population has remained unclear.

Few studies in rodent models of SCI have examined the role of age after trauma. Aged male rats exhibit greater locomotor and reparative deficits than adult and/or young rats following hemisection [5], clip compression [6], and contusion SCI [7]. Critically, although females make up 39% of individuals living with paralysis due to a traumatic spinal cord injury [8], few studies have addressed age-related effects on recovery and myelin pathology after SCI in female rats [9,10]. Further, age-associated differences in gene expression of some inflammatory components have recently been identified but the effect of transcription changes on immune-related protein synthesis has not been investigated [11]. As well, while an effect of age on some components of cellular inflammation after SCI has been suggested in early development [12], changes in inflammatory responses have not previously been assessed in young versus aged animals. Finally, age-related changes in pathology have been based primarily on conventional morphometric analysis of postmortem spinal cord tissue. However, this type of analysis does not address volumetric changes in the spinal cord due to either expansion or compression of tissue post-SCI, or differences associated with normal aging. Interestingly, while the impact of age-related structural changes of the brain on quantification parameters have been highlighted [13], there has been an overall paucity of studies accounting for size changes in the aging spinal cord. Recently, a single study investigating this issue in uninjured spinal cord demonstrated age-associated morphometric changes [14], emphasizing the importance of volume-corrected quantification.

In the present study, we used a clinically relevant SCI model to characterize the role of age on recovery, repair, and inflammatory processes, focusing on the innate cellular and humoral immune response.

## Results

### IH device-generated contusion injuries are similar in aged and young rats

The Infinite Horizon (IH) Impactor was used to generate contusion injuries. This device has been shown to induce reproducible graded injuries in rodents [15,16]. Biometrics of the injury are monitored by a force-driven sensor, measuring both the actual force and the resulting displacement delivered to the spinal cord [15]. Although differences in total body weight were observed between the young and aged cohorts, no significant differences between groups in either force or displacement was detected (Table 1), suggesting that lesion parameters were comparable across age.

**Table 1:** IH device-generated contusion injuries in aged and young rats

	**Weight (g)**	**Actual force (kd)**	**Displacement (μm)**
Young	190 + 2.70	208 + 1.77	1425 + 72.9
Aged	265 + 3.84*	209 + 1.88	1349 + 71.7

The weight and corresponding biometrics of contusion injuries for the animals used in these studies are shown above. Aged rats weighed significantly more than their younger counterparts (Student’s t test, *p<0.05). However, despite this difference, no statistically significant differences were observed in the actual force and resulting displacement of aged versus young spinal cord tissue (Student’s t test, p >0.05). Mean ± SEM is shown.

### Aged rats showed greater locomotor deficits than their young counterparts

**Figure 1:**
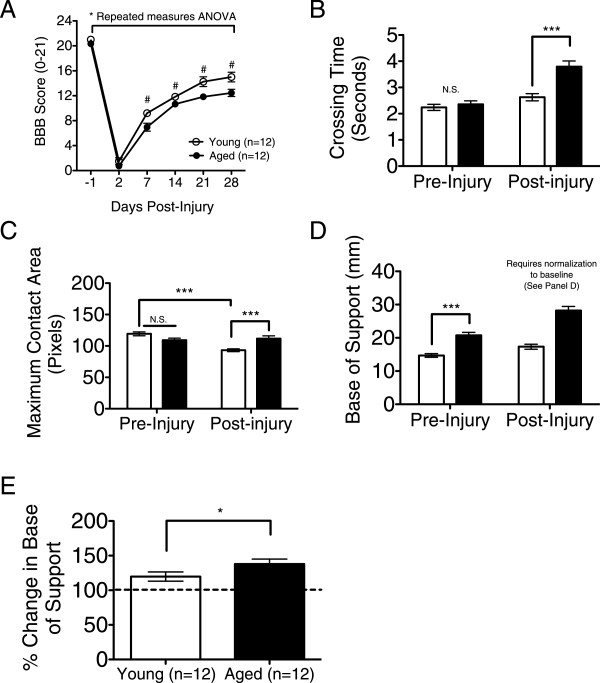
**Aged rats showed greater locomotor deficits. (A)** Repeated-measures ANOVA yielded a main significant effect for performance of young vs aged animals (F = 2.36, *p = 0.044) in the open-field. Post-hoc tests revealed a significant difference between age groups at 7, 21 and 28 days post-injury (Bonferroni’s multiple comparison, #p<0.05). On day 28, aged rats showed greater locomotor deficits compared to the young. **(B)** Using CatWalk analysis, no differences in crossing time were found between age groups at baseline, but post-SCI, aged animals demonstrated an increase in this parameter compared to young. **(C)** Young animals exhibited decreased contact area compared to aged animals post-injury as well as relative to their pre-injury baseline. **(D-E)** Since base of support was significantly different between age groups at baseline **(D)**, post-injury data were normalized to pre-injury performance (dashed line in **E**) and demonstrated a significant increase in base of support of aged animals compared to young **(E)**. Student’s t-test were used to compare between groups: ***p< 0.001; **p< 0.01, *p<0.05. Mean ± SEM is shown.

Recovery of locomotor function was assessed by the BBB open-field locomotor scale prior to injury, as well as post-injury on days 2, 7, and weekly thereafter for 4 weeks. Repeated-measures ANOVA demonstrated a significant difference between young versus aged animals (Figure 1A). Post-hoc tests revealed a significant group difference at 7, 21, and 28 days post-injury (dpi). At 28 dpi, young and aged rats achieved a final average score of 15 (consistent plantar stepping with consistent coordination) and 12.5 (frequent to consistent plantar stepping with occasional coordination), respectively. These data suggest that gross locomotor recovery in aged animals was significantly impaired relative to young.

To further investigate recovery of fine parameters of gait, CatWalk analyses were performed. All animals were tested prior to injury and terminally at 28 dpi; only measures in which a significant change between the two age groups was observed are reported. No difference in crossing time between groups was found prior to SCI, but a significant increase in crossing time of aged animals was observed post-injury, suggesting impairment in movement kinetics (Figure 1B). Surprisingly, aged animals exhibited an increase in contact area compared to the young post-injury (Figure 1C) while young animals showed decreased contact area relative to their pre-injury baseline. Max paw contact is defined as the area contacted at the moment of maximum paw-floor contact during stance phase [17]. A decrease in max contact area can reflect avoidance of complete placement due to altered sensitivity [18]. In addition to these measures, base of support was evaluated as a reliable indicator of locomotor recovery following rat contusion SCI. Base of support was significantly different between young and aged animals at baseline (Figure 1D); therefore, post-injury numbers were normalized to pre-injury performance for each animal. While both young and aged animals revealed an increase in base of support post-injury compared to baseline (dashed line in Figure 1E), this change was greater in aged than young. As well, aged animals demonstrated a significantly wider base of support compared to young (Figure 1E), suggesting compensation for trunk instability. Altogether, change in walkway crossing time and base of support suggest decreased locomotor recovery in aged rats.

### Lesion area is greater in aged than young animals immediately caudal to the epicenter

**Figure 2:**
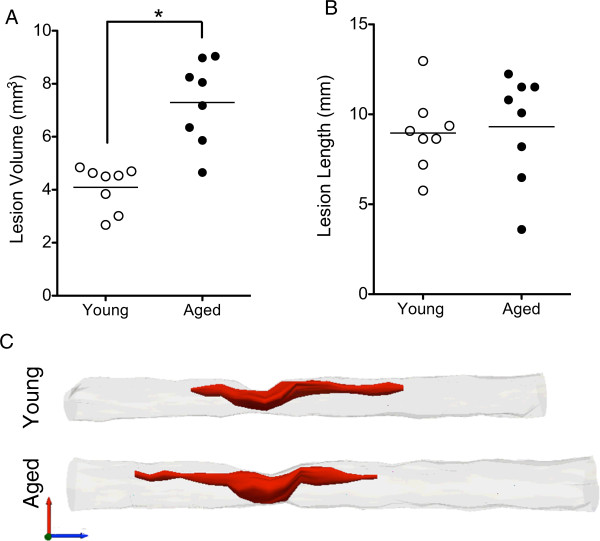
**Morphometric tissue analysis revealed the critical importance of volume correction.** Using direct comparisons, aged rats showed a significant increase in volume of injured tissue compared to their young counterparts (Student’s t test, *p< 0.05**) (A)**. No age-associated differences were observed in the total lesion spread **(B)**. Three-dimensional reconstructions of representative injured spinal cord segments (T6-T12) suggested increased length and cross-sectional area in aged animals compared to young **(C)**.

Traumatic injury to the spinal cord results in extensive tissue damage that spreads rostral and caudal to the injury. A prominent pathological feature of SCI in rats is the development of a fluid-filled cystic cavity that is bordered by reactive astrocytes [19,20]. To investigate whether age influenced lesion volume, the cavity was identified by loss of cells and abnormal cytoarchitecture using Nissl staining, and quantified using unbiased stereology. Aged rats exhibited a significant increase in total lesion volume (7.2 ± 0.56 mm^3^) compared to young (4.1 ± 0.30 mm^3^) (Figure 2A). However, the extent of lesion spread (measured as total lesion length), was not significantly different between groups (aged = 9.4 ± 1.1 mm; young = 9.1 ± 0.77 mm) (Figure 2B).

**Figure 3:**
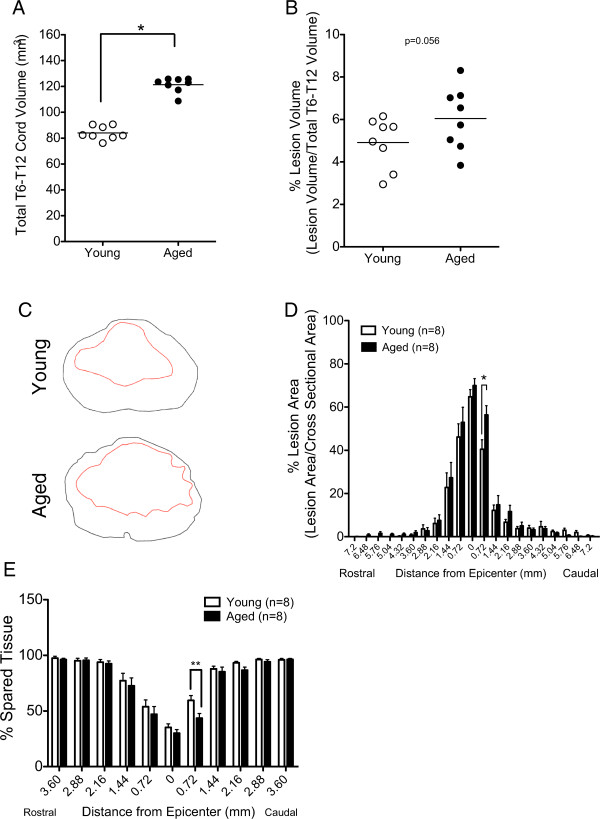
**Volume correction demonstrated increased and localized pathology in aged rats compared to young at 28 dpi.** Confirming observations in 3D reconstructions, stereological assessment of total spinal cord volume between T6 and T12 dorsal roots revealed a significant difference between age groups (Student’s t test, *p< 0.001) **(A)**. Accordingly, lesion volume from Figure 3A was normalized to total cord volume **(A)** and expressed as a percentage. As such, aged rats showed a strong but non-significant trend for increased % lesion volume compared to young rats (Student’s t test, p= 0.056) **(B)**. Stereologically analyzed coronal diagrams from a single section immediately caudal to the injury epicenter demonstrated potential differences in lesion area (red line) relative to cross-sectional area (black line) **(C)**. When lesion area at each cross-section was normalized to total area at the same cross-section and quantified along the length of the spinal cord, a single point of significance was detected immediately caudal to the injury epicenter, where % lesion area of aged animals was significantly greater than the young (Student’s t test, *p< 0.05) **(D)**. **(E)** Additionally, quantification of the % spared tissue along the length of the spinal cord demonstrated the same effect as % lesion area, confirming localized differences between age groups in pathology (Student’s t test, *p< 0.05). Data points represent individual animals and the horizontal line indicates group mean.

Notably, two key issues related to volume analyses must be considered: 1) the effect of injury on tissue shrinkage/swelling, which may differ across age groups, and 2) the effect of age on the overall size of the spinal cord. As evidenced by the 3D reconstruction of the lesion and in naive animals (Figure 2C), aged animals have both greater length (aged = 24.6 mm ± 0.24 vs young = 22.6 mm ± 0.3) and greater cross-sectional area (aged = 4.92 mm^2^ ± 0.082 vs young = 3.72 mm^2^ ± 0.068) compared to young. This translates to an 8% increase in length and a 25% increase in cross-sectional area in aged spinal cords compared to young, thus resulting in an increase in total cord volume (Figure 3A). Accordingly, it is critical to correct for the resulting difference in the total volume. We therefore expressed lesion volume as percentage of total volume (% lesion volume). Comparison of % lesion volume revealed a strong trend toward an increase in aged animals (p = 0.056; Figure 3B).

However, cross-sectional analysis suggested possible differences in lesion-mediated disruption of descending white-matter tracts (Figure 3C). Thus, the % lesion area (red line) per cross-sectional area (black line) was analyzed. These data demonstrated a specific and localized age-associated increase in lesion area at regions immediately caudal to the injury epicenter (Figure 3D) and demonstrate that development of lesion pathology is age-dependent, suggesting greater disruption of ascending and descending white matter tracts in aged animals. This provides a potential mechanism for locomotor differences between age groups. In parallel, recent reports have identified age-associated attenuation of CST and 5-HT fiber sprouting in a region-specific manner, suggesting differential effects on motor outcome [21].

### Less tissue is spared caudal to the epicenter in aged animals compared to young

In parallel to the findings demonstrating increased lesion size in aged versus young rodents, analysis of the amount of spared tissue revealed a similar trend. Significantly more tissue sparing was observed in young animals immediately caudal to the injury epicenter compared to old (Figure 3E). These data suggest preservation of projection pathways below the level of injury that could underlie increased locomotor recovery in young rats.

### Acute cell death distal to the lesion is greater in aged than young animals

**Figure 4:**
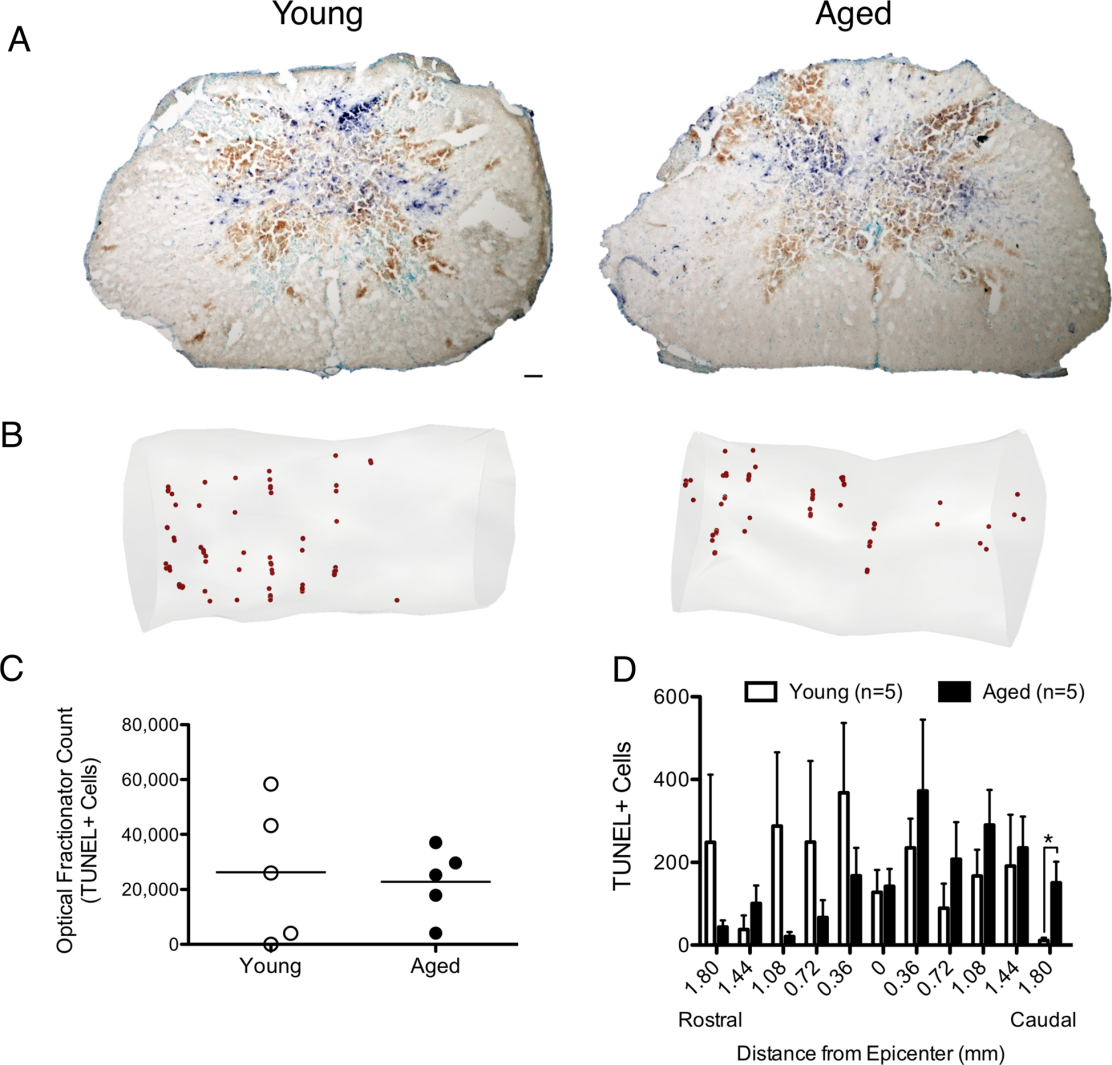
**Early TUNEL-mediated cell death was selectively increased caudal to the epicenter in aged animals.** Low power representative photomicrographs of coronal spinal cord sections from aged and young animals labeled for TUNEL (blue) and counter-stained with methyl green (green) are shown **(A)**. 3D reconstructions of TUNEL-positive quantification revealed a heterogeneous distribution of TUNEL-positive cells in both age groups **(B)**. Stereological quantification of TUNEL-positive cells showed no significant differences between the total number of TUNEL-positive cells in aged and young animals **(C)**. Detailed distribution analysis of TUNEL-positive cells demonstrated a single point of significance where in the caudal spinal cord region, significantly more apoptotic cells were observed in aged animals compared to the young (Student’s t test, *p< 0.05) **(D)**. Scale bar = 100 μm.

It has been previously reported that cell loss shortly after SCI contributes to lesion expansion and ultimately cavity formation [22]. In order to characterize early cell death, TUNEL-positive nuclei were quantified. At 2 dpi, the injury epicenter revealed extensive hemorrhaging as noted by the large number of red blood cells in both groups (brown) (Figure 4A). Stereological 3D reconstructions of representative animals suggested similar overall numbers of TUNEL-positive cells (Figure 4B). Stereological quantification showed no significant differences in total cell number between groups (Figure 4C). However, in parallel with % lesion area, aged animals demonstrated an increase in TUNEL-positive cells caudal to the epicenter (Figure 4D). These data suggest that age may impact acute cell death in a region-specific manner.

### Early and localized inflammatory response in aged animals is significantly higher than young

As noted, the effect of age on inflammatory processes following SCI has not been extensively investigated, even though innate immune activation has been shown following SCI [23-28]. Thus, we investigated whether young and aged animals exhibited differences in either the humoral or cellular components of the inflammatory response.

**Figure 5:**
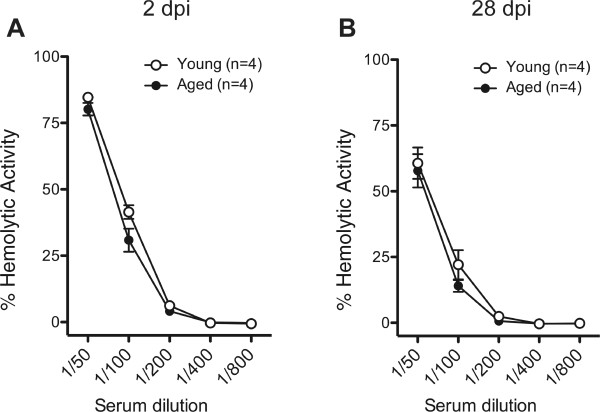
**Total systemic complement activity after SCI was not influenced by age.** CH50 assays showed that systemic complement activity in young animals was not significantly different from that of aged animals at 2 days post-SCI **(A)** or at 28 days post-SCI **(B)** (Student’s t test, p >0.05). Mean ± SEM is shown.

**Figure 6:**
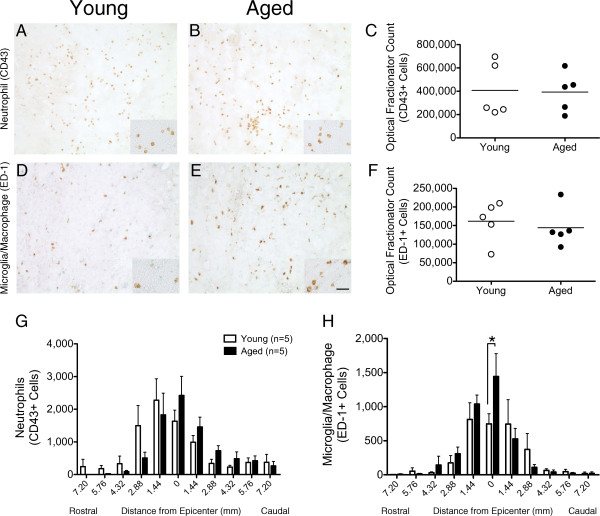
**Aged rats showed an acute and localized increase in microglia/macrophage infiltration.** Representative photomicrographs of coronal spinal cord sections immunolabeled for neutophils with CD43 **(A-B)** or microglia/macrophages with ED-1 **(D-E)** and counter-stained with methyl green are shown. Insets are of individual fields at higher magnification. Unbiased stereological quantification of total numbers of CD43-positive cells **(C)** and ED-1 positive cells **(F)** revealed no significant differences between age groups. Rostro-caudal distribution of CD43+ cells did not exhibit differences between groups (p > 0.05) **(G)**. In contrast, ED-1 positive cells showed significantly increased age-associated microglia/macrophage response at the injury epicenter **(H)**. Student's t-tests, *p< 0.05. Mean ± SEM is shown. Scale bar = 25 μm.

Complement activation has been implicated in mediating cell death through generation of the membrane attack complex (MAC) [29,30]. We have previously shown immunoreactivity for C5b-9 in association with neurons and oligodendrocytes as early as 24 hours and extending through 42 days after SCI [28], as well as systemic complement activation after SCI via CH50 assay of hemolytic activity [27]. Here, we investigated whether age influenced systemic complement activation post-SCI. Erythrocyte lysis post-SCI was confirmed, however, hemolytic activity in aged animals was not significantly different from young at 2- (Figure 5A) or 28- (Figure 5B) dpi.To investigate whether age influenced the acute cellular inflammatory response, the magnitude and spatial distribution of neutrophils and microglia/macrophages was quantified. At 2 dpi, the injury epicenter showed extensive CD43 (neutrophils) and ED-1 (microglia/macrophages) labeling (Figure 6A-B, D-E). Stereological analyses revealed no differences in cell numbers between groups for either cell type (Figure 6C, F). As well, the distribution of neutrophils was not different between aged and young rats at either the injury epicenter or distances rostral and caudal to the lesion (Figure 6G). In contrast, significantly higher numbers of ED-1-positive cells were observed in aged rats at the injury epicenter compared to young (1443 ± 334.6 versus 747.7 ± 148.4, respectively) (Figure 6H). Concomitant with previous observations demonstrating increased lesion area near/immediately caudal to the epicenter, the increase in macrophage/microglial response may indicate evidence of increased phagocytosis/debris clearance in aged animals.

## Discussion

According to a number of prospective studies on the prevalence of paralysis in the US [8,31], the average age at time of spinal cord injuries (SCI) in the clinical setting is 34 and rising. Of significant importance to the clinical population, age at the onset of injury influences disability [31] and is linked to inferior trauma outcomes [32]. Furthermore, the incidence of traumatic SCI in older adults increased from 79.4 per million older adults in 2007 to 87.7 by the end of 2009, but remained steady among younger adults, suggesting a large and growing clinical demand [32]. However, most animal models of CNS injury primarily use young rodents. Critically, understanding the mechanisms of recovery and repair in an aging model of SCI could provide significant insight for the potential therapeutic efficacy of intervention strategies in the human population. The present study is the first to investigate the effects of age on a number of parameters associated with behavioral recovery, repair processes, and the inflammatory response after contusion SCI in female rats. We report that, consistent with studies in male animals, aged female rats demonstrated greater locomotor deficits compared to young. Surprisingly, young but not aged rats exhibited altered hindlimb sensory function.

Both experimental animal studies and clinical cases have shown that SCI initiates an acute innate cellular and humoral inflammatory response that is characterized by infiltration of neutrophils, microglia/macrophages [12,24,26,27,33,34], and the complement cascade [27,28,35]. In the aging CNS, both primate and human clinical studies have shown increased levels of complement activation products in the brain of aged versus young subjects [36]. However, in the present study, no differences in early (2 dpi) systemic levels of complement activation were detected between age groups, suggesting that this aspect of the inflammatory response did not contribute to age-associated differences in lesion pathogenesis.

In parallel, stereological analyses demonstrated no differences in neutrophil number between aged and young female rats. This observation is in contrast to a previous study in male rats following clip compression SCI [6]. Although, we cannot rule out the possibility that differences in injury model and sex may influence neutrophil infiltration, the discrepancy between these studies may also be attributed to the methodology used to assess neutrophil infiltration. While authors of the previous study used myeloperoxidase (MPO) activity to assess neutrophil influx, we used unbiased stereology to quantify CD43-positive neutrophils in the spinal cord. Although assessment of MPO activity is often used as an indirect measure of neutrophil infiltration in tissue, this technique is not a quantitative measure of cell number. Furthermore, microglia/macrophages have also been shown to produce MPO [37,38]. Thus, previous reports of age-associated increase in MPO activity [6] could be at least partly attributed to elevated microglial/macrophage response, which would be consistent with the findings reported here.

While elevated numbers of microglia/macrophages have been shown in a number of different animal models of CNS injury [39-41], to date, only one group has attempted to evaluate the effect of age on microglial production of pro- and anti-inflammatory cytokines after SCI and suggest an age-associated increase in pro-inflammatory processes [12]. However, an exceptionally narrow range between age groups (4 weeks versus 10 weeks) limits the interpretation of these results. The data presented here compare 3- and 18-month old female rats, a much more relevant range in the context of aging, and consistent with previous studies performed in CNS injury paradigms, these data show increased microglia/macrophage infiltration at the injury epicenter.

Mechanistically, both detrimental [23,25,42,43] and beneficial [44,45] functions have been attributed to macrophages following SCI. The dichotomy in macrophage/microglia function has been associated with a number of factors including location, timing, and activation state. Histological studies of spinal cord tissue have shown that the location of macrophage infiltration (i.e. proximal to the injury epicenter versus the intact parenchyma) can reflect pro- or anti-inflammatory phenotypes [46,47]. Macrophages/microglia are present in large numbers in areas of necrosis, likely mediating phagocytosis of debris, and to a lesser extent in regions rostral and caudal to the epicenter in which the cryoarchitecture appears normal. Furthermore, we have previously shown a biphasic ED-1 response at acute and chronic phases of SCI and demonstrated locomotor decrements when the delayed inflammatory response was blocked, suggesting that the early inflammatory response attenuates injury [27]. Finally, the duality in the activation states of infiltrating cells (i.e. M1 versus M2 phenotypes) has been shown: classically activated M1 macrophages are neurotoxic and contribute to tissue destruction while the M2 subtype are reparative and promote axonal regeneration [46-48]. Interestingly, the acute macrophage response following SCI (within the first 3 days) is predominantly associated with M1 macrophages. Altogether, these data are consistent with our findings and suggest that the early and localized increase observed in ED-1-positive cells attenuates injury and may explain the increase in TUNEL-positive cells and the increase in lesion size immediately adjacent to this region.

We also report a decrease in paw contact area in young animals following SCI. Notably, reduced paw intensity has been correlated with decreased von Frey withdrawal threshold, suggesting that CatWalk gait analysis could be utilized as a supplemental tool to measure mechanical allodynia [18]. Traumatic injury to the spinal cord can cause aberrant sprouting of sensory pathways that may result in the development of chronic central pain syndromes such as autophagia and allodynia [49,50]. While evidence for an increase in mechanical allodynia has been reported in young versus old animals receiving unilateral hemisection injuries [5], contusion injuries using the IH Impactor fail to elicit the development of mechanical allodynia in a large percentage of animals unless there is a dwell time associated with the injury force or if the force is severe [51]. However, the current data suggest that young rats may exhibit greater sensory alterations than aged rats after SCI.

## Conclusions

In summary, these data provide novel insight into the pathology and acute immune response in aged and young animals following contusion SCI and extend previous findings regarding the role of age in repair processes. While gender-specific hormones, especially estrogen, may also affect pathophysiology, our understanding of these mechanisms is limited and data suggest a highly complex interplay in this context [9,52-55]. Importantly, the results of the present study are consistent with previous experiments demonstrating locomotor deficits in a variety of SCI male models [5-7], strongly suggesting that age alters recovery. Ultimately, understanding age-associated mechanisms of recovery and repair will be instrumental in designing therapies aimed at improving function in the clinical population.

## Methods

All experiments and surgical procedures were conducted in accordance with the Institutional Animal Care and Use Committee guidelines at UCI.

### Subjects

Young (3 months of age: 15 weeks old and all born on the same day) (n = 20) and aged (18 months of age and all born on the same day) (n = 20) female Fisher 344/Brown Norwegian F1 hybrid rats were obtained through the National Institute of Aging in Harlan Indiana. This colony of rats was selected because unlike other inbred strains (i.e. Sprague Dawley), Fisher 344 rats do not develop kidney disease, cancer and other known confounding problems that are often times associated with aging (Personal communication with NIA). Prior to surgery, animals were randomly assigned into the following groups: 2 day survival cohort for histological/stereological assessment of inflammatory cells (n = 8 per group) and a 28 day survival cohort for behavioral analysis and histological/stereological assessment of lesion volume (n = 12 per group). Prior to surgery, all rats were handled daily for 2 weeks.

A total of 4 animals from the 2 day cohort was excluded based on: spinal cord bruise during laminectomy (n = 1; aged), confirmed under a dissection microscope; abnormal time versus force curve, indicating bone hit (n = 1; aged); incorrect spinal cord root dissection (n = 1; aged); and high 2d BBB score, indicating abnormal recovery (n = 1; young). There was no mortality due to or following surgery for either group. The final n’s were: young (n = 7) and aged (n = 5) for 2 day cohort and young (n = 12) and aged (n = 12) for 28 day cohort.

### Surgical procedures

Sterile surgical techniques were used. Rats were anesthetized with a mixture of 80% Ketamine (100 mg/kg) and 70% Xylazine (10 mg/kg). Spinal cord was exposed at thoracic vertebrae 9 (T9) by laminectomy. Rats received a 200 kilodyne (1 dyne=10μN) contusion injury with the Infinite Horizon Impactor (Precision Systems and Instrumentation), and a small piece of Gelfoam was placed over the laminectomy, musculature sutured and skin reconnected using metal wound clips. Body temperature was monitored and all animals were housed in pairs, placed on water-jacketed heating pads overnight, and returned to their housing room the next day.

All rats received subcutaneous injections of Lactated Ringers (5 ml/100 g) for 3–5 days following SCI and thereafter if necessary; Baytril (2.5 mg/kg) was administered during the first 7 days of the study. Additionally, all rats received Buprenorphine (0.01 mg/kg) every 12 hours for 48 hours post-SCI. Manual bladder care was performed for 2 weeks post-injury, 2 times a day or until the animal regained complete bladder function.

### Behavioral assessments

Prior to SCI, 2 dpi, 7 dpi, and on a weekly basis thereafter for 4 weeks, 2 independent observers blinded to experimental groups used the open-field locomotor scale to assess spontaneous recovery.

CatWalk Gait analysis (CatWalk 6.13 software, F. Hamers)- also evaluated by observers blinded to the groups- was used to assess gait dynamics pre-SCI and at the end of the study, day 28. The following criteria were employed in all acquisitions: (1) the crossing was uninterrupted and at a consistent pace, (2) a minimum of three crossings was performed per animal (resulting data was averaged for statistical analysis).

### Hemolytic complement assay (CH50)

A total of 4 animals per age group per cohort was randomly selected for assessment of serum complement activity. Prior to perfusion, 0.5-1 ml of blood was withdrawn by cardiac puncture and collected in borosilicate glass tubes and allowed to clot. After centrifugation of the sample at 2000 rpm, serum was recovered and stored at -80°C until assayed for complement activity. Total complement activity was tested as previously described [56] by adding 50 μl (50 × 10^8^/ml) of sensitized erythrocytes (EA), prepared by incubation of sheep erythrocytes (E) (Colorado Serum Co.) with rabbit anti-sheep red blood cell antibodies (A’s, 1/75 IgG), to serial dilution of the serum (1/15, 1/30, 1/60, 1/00) diluted in 250 μl of Gelatin Veronal Buffer (GVB^++^) on ice. Replicate reaction mixtures were prepared. Samples were incubated at 37°C, and then centrifuged at 3000 rpm to pellet un-lysed erythrocytes. Supernatants from replicate reactions were placed in 96-well plates, and the optical density (OD) was measured at 405 nm. Percent of total complement lytic activity correspondent to the percentage of EA’s lysis was calculated: [(sample OD)-(buffer control OD)]/[(H_2_0 lysis OD)-(buffer control OD)].

### Tissue collection

Rats were anesthetized with sodium pentobarbital (0.4 ml) and euthanized by intracardiac perfusion with 4% paraformaldehyde. Spinal cord segments were dissected by T6-T12 spinal roots, sunk in 20% sucrose/4% paraformaldehyde overnight, flash frozen in isopentane (2-methlybutane), and stored at -80°C.

### Immunohistochemistry for CD43 and ED-1

Spinal cord segments from the 2 dpi survival cohort were cut coronally (30 μm) on a cryostat, collected on gelatinized (Sigma-Aldrich) slides and stored at -20°C until needed for immunohistochemistry. For stereological quantification, a set of slides containing a complete series of parallel sections (every 24^th^) from the entire injury segment of the spinal cord (T6-T12) was processed as described below. Briefly, every 24^th^ section from the 2 day survival cohort was processed for CD43 (1:1,000; Serotec MCA54G), or ED-1 (1:2,000; Serotec MCA341R). Sections were incubated with the appropriate species-specific affinity-purified antibodies followed by avidin-biotin complex and visualized with diaminobenzidine (DAB) (Vector Labs). Control sections were incubated without primary antibody to test for antibody specificity.

### Nissl staining

Spinal cord segments from the 28 day survival cohort were cut and stored as described above. Briefly, the slides were rehydrated for 40 min in 0.1 M Tris (pH 7.4). Slides were then washed for 10 min in Tris/0.1% Triton for tissue permeabilization, washed twice for 5 min in Tris, and then 250 μl of fluorescent Nissl neurotracer (1:20, Invitrogen) was added to the sections and incubated in the dark for 20 min. Unbound stain was removed with Tris/0.1% Triton. Slides were washed three times and cover-slipped with Prolong gold antifade reagent with DAPI nuclear counterstain (Invitrogen).

### TUNEL labeling

TUNEL labeling was performed according to the manufacturer’s instructions with slight modifications (Millipore). Briefly, a set of slides from the 2 day cohort was hydrated in Sodium Citrate buffer (10 mM, pH 6.0) for 5 min at RT and then boiled in fresh Sodium Citrate buffer for 5 min. Slides were washed 2X in PBS prior to incubation with 20 μg/ml of proteinase K (Sigma) for 5 min at RT to remove nuclear proteins. Slides were washed 2X in PBS, then incubated in equilibrium buffer for 10 min. Excess equilibrium buffer was removed and slides were then incubated with TdT and dUTP-digoxigenin at 37°C for 1 hr in a humidified chamber. To stop the reaction, slides were placed in stop/wash buffer for 15 min at RT and then washed 2X in PBS before incubating the slides with anti-digoxigenin-alkaline phosphatase conjugated antibody (1:500) for 1 hr at RT. Excess antibody was removed by washing the slides with PBS 4X. Slides were visualized with BCIP and NBT (Roche) substrate and counter-stained with methyl green. Control sections were processed as described above except that either the TdT-dUTP-digoxigenin reaction mixture or the anti-digoxigenin-alkaline phosphatase conjugated antibody were omitted.

### Stereological quantification

Unbiased stereological analysis was performed on a series of coronal sections (35–40 sections per animal) using the StereoInvestigator system (MicrobrightField, version 7.003). All analyses were performed using an Olympus BX51 microscope with a motorized stage under identical light microscopy conditions for each of the parameters measured. Starting sections were chosen randomly and every 24^th^ section throughout T6-T12 was analyzed. The grid sizes used were determined empirically to arrive at a coefficient of error (CE) that was <0.10 for most parameters examined (Table 2). The only exception was TUNEL quantification where due to the small presence and spread of cells, even when setting up grid size and counting frame sizes that were predicted to over-sample total cell numbers, CE values below 0.15 could not be obtained. All stereological analyses were performed by individuals blinded to experimental groups.

**Table 2:** Intra-animal variation in stereological analyses

	**Average mean coefficient of error**	
**Immunohistochemical parameter**	**Young**	**Aged**
Total cord volume	0.005	0.004
Lesion volume	0.026	0.021
TUNEL positive cells	0.143	0.133
ED-1 positive cells	0.044	0.048
CD43 positive cells	0.072	0.066

The Cavalieri Estimator was used at 10X to quantify lesion volume, area, area of spared tissue, and total cord volume and area in Nissl-stained sections. The lesion was defined as the area that contained cavitation (complete absence of tissue) and those areas that contained abnormal cytoarchitecture. Area of spared tissue was then determined by subtracting the lesion area from the total area. Percentage of lesion and area were calculated as follows: (Lesion area or area of spared tissue/Total cord area) × 100.

The optical fractionator was used as previously described to quantify the number of CD43-positive and ED-1-positive cells. Sections immuno-labeled with ED-1 were counterstained with methyl green. Analyses were carried out using a grid size of 200 μm × 200 μm and a corresponding counting frame of 50 μm × 50 μm. Quantification of CD43-positive and ED-1-positive cells was carried out using 60X and 100X objectives, respectively. The optical dissector height was 8 μm and the upper guard zone was set at 2 μm.

The mean and p-value results for each of the stereological parameters are summarized in Table 3.

**Table 3:** Mean and p-values for each of the stereological parameters

	**Mean values**		**p-value**
**Immunohistochemical parameter**	**Young**	**Aged**	
Total cord volume	84.10	121.3	< 0.0001
Lesion volume	4.092	7.294	< 0.0001
% Lesion volume	4.92%	6.05%	0.056
% Spared tissue at 0.72 mm Caudal	56.41%	40.49%	0.0101
TUNEL positive cells	26,328	22,811	0.7860
ED-1 positive cells	161,514	144,251	0.3123
CD43 positive cells	408,183	393,057	0.3055

### Statistical analysis

Comparisons between groups on the BBB locomotor rating scale were performed using repeated measures ANOVA with post-hoc Bonferroni Dunn (Prism; Graphpad). Student’s *t*-tests were used for group comparisons of data obtained from CatWalk, CH50 assays and stereological analyses. Values of p ≤ 0.05 were considered significant. All data are expressed as mean ± standard error mean (SEM).

## Competing interests

The authors declare that they have no competing interests.

## Authors’ contributions

MJH, MDG, and AJA were responsible for study design. MJH and MDG were responsible for behavioral data acquisition and analysis. MJH and MDG were responsible for histology data acquisition. MJH, MDG, and EP were responsible for stereological analysis. MJH and MDG were responsible for data interpretation. MJH and MDG were responsible for drafting the manuscript. MJH, MDG, EP, and AJA were responsible for manuscript revision and final approval for submission.
